# Recognizing Non-Collaborative Radio Station Communication Behaviors Using an Ameliorated LeNet

**DOI:** 10.3390/s20154320

**Published:** 2020-08-03

**Authors:** Zilong Wu, Hong Chen, Yingke Lei

**Affiliations:** College of Electronic Countermeasures, National University of Defense Technology, Hefei 230037, China; wuzilong@nudt.edu.cn (Z.W.); ch2sun@mail.ustc.edu.cn (H.C.)

**Keywords:** communication behaviors, bispectrum estimation, signal recognition, convolutional neural network (CNN), short-wave radio station

## Abstract

This work improves a LeNet model algorithm based on a signal’s bispectral features to recognize the communication behaviors of a non-collaborative short-wave radio station. At first, the mapping relationships between the burst waveforms and the communication behaviors of a radio station are analyzed. Then, bispectral features of simulated behavior signals are obtained as the input of the network. With regard to the recognition neural network, the structure of LeNet and the size of the convolutional kernel in LeNet are optimized. Finally, the five types of communication behavior are recognized by using the improved bispectral estimation matrix of signals and the ameliorated LeNet. The experimental results show that when the signal-to-noise ratio (SNR) values are 8, 10, or 15 dB, the recognition accuracy values of the improved algorithm reach 81.5%, 94.5%, and 99.3%, respectively. Compared with other algorithms, the training time cost and recognition accuracy of the proposed algorithm are lower and higher, respectively; thus, the proposed algorithm is of great practical value.

## 1. Introduction

In the field of electronic counter-measures, only physical layer signals can be detected by sensors. Therefore, research on the communication behavior of radio stations must be carried out by analyzing the physical layer signals. In the absence of communication protocol standards, as a non-collaborator, correctly recognizing communication behaviors has always been a difficult problem [1,2]. The communication behavior of a radio station represents the working state of the radio station, which helps us to infer the communication intention of the radio station’s holder. It is of great significance to carry out research on communication behaviors by directly using physical layer signals detected by sensors.

The communication behavior of a short-wave radio station refers to the behavior generated by the targeted radio station, which transmits voice, data or images. Communication behaviors include “link establishment–link demolition”, “service request–service confirmation”, and “service transmission”. In this work, communication behaviors of a short-wave radio station are divided into five categories: automatic link establishment (ALE) behavior, traffic management and high-rate data link protocol (HDL) acknowledgement (TMHA) behavior, HDL traffic data (HTD) behavior, low-rate data link protocol (LDL) traffic data (LTD) behavior, and LDL acknowledgement (LA) behavior. The five kinds of communication behavior correspond to five kinds of burst waveforms. If we can distinguish the kind of burst waveform (BW) a radio station sends, we can establish the radio station’s communication behavior, and at the same time we can determine the radio station’s working status. Mastering the working status of the radio stations of non-collaborative organizations can provide us with more intelligence information, which is very difficult if there is no communication protocol standard. The traditional recognition methods for a radio station’s communication behavior need to go deeply into the framework in the data link layer, which is not possible in real electronic countermeasures. In this work, the analysis of communication behaviors is carried out directly based on the physical layer signals, which are able to be collected by sensors in the real environment. A novel algorithm for radio station communication behavior recognition is proposed.

There is almost no existing research on non-collaborative radio station communication behaviors due to many difficulties in the field. Previous studies [3,4] explored communication relationships between radio stations by analyzing the intercepted radio signals. However, these communication relationships are not our focus. However, Wu et al. [5] proposed a method for radio station link establishment (LE) behavior recognition, and the method can recognize seven kinds of LE behaviors without a communication protocol. The seven types of LE behavior would be categorized as ALE behavior in this work. In fact, there are five kinds of radio station communication behavior, which means the LE behavior recognition proposed by Wu et al. [5] differs from the communication behaviors studied in this work. There are also other methods; for example, the flow mining (FlowMine) algorithm was proposed to mine the instruction flow and was verified with simulated and real data [6]. Another study [7] completed feature selection and data classification for binary protocol packets, which facilitated further study of the communication protocol recognition. Moreover, the FlowMine conversation relationship extraction method—which is based on conversation revivification and protocol identification methods, combing port and regular expressions—was proposed to quickly locate Fetion service packets in promiscuous raw packets to achieve various business relationships [8]. However, in those studies [6,7,8], the main focus of research was the identification of communication relationships or a communication protocol, while the research on communication behaviors of specific targets is not in-depth. These previous studies [6,7,8] may infer communication behaviors represented by the physical layer signals by analyzing the communication protocol standards, but this is difficult to apply without a communication protocol standard. Since we cannot demodulate and decrypt correctly, we must break through the limitations of the communication protocol, which is impossible as a non-collaborator.

According to a simple analysis of the communication behavior signals of a short-wave radio station, the five types of communication behavior signals corresponding to five kinds of burst waveforms differ slightly in their durations, which are specified in the third-generation short-wave communication protocol standard called military standard-188-141B (MIL-STD-188-141B). However, in the real world, it is difficult to distinguish between the small differences in different burst waveforms. In this work, a bispectral feature transformation is used to transform signals into matrices with the same dimensions that are easily and conveniently imported to a neural network. Bispectral feature transformation is widely used in signal recognition. For example, Han et al. [9] and Cao et al. [10] used bispectral features of signals to carry out signal classification and Wang et al. [11] used bispectral diagonal slices of signals to make extracted features of signals simpler and more obvious. After bispectral feature transformation, the signal can be directly recognized by the classifier, but recognition performance is often not ideal. In addition, a convolutional neural network (CNN) can extract the deeper features of the signal bispectrum [12,13,14,15]. In this case, after the physical layer signals are collected by sensors, the method of signal bispectrum estimation combined with CNN can infer the communication behaviors of a radio station. Generally speaking, the more complex the CNN, the better the network performance. The first classic architecture of CNN was the network proposed by Yann LeCun called LeNet. Even though it has existed for many years, it is still widely used in fault diagnosis and gas identification [16,17]. The more complicated network proposed by Alex Krizhevsky called AlexNet appeared later, but it does not differ much from LeNet in architecture [18,19]. As far as network architecture is concerned, a network proposed by Google called GoogLeNet, residual network (ResNet), and densely connected convolutional network (DenseNet) are all innovative. In addition, GoogLeNet [20,21] and ResNet [22,23,24] are used widely in hand gesture recognition, image retrieval, and visual recognition. DenseNet can reuse the features of initial samples and has better performance than other CNN models. Therefore, DenseNet is widely utilized in speech recognition, disease diagnosis, and detection of wildfire smoke images [25,26,27]. To speed up application, LeNet was employed to conduct the extraction of features and recognition of communication behaviors because it is the simplest method.

The recognition of communication behaviors of a non-collaborative radio station by directly analyzing physical layer signals without using a standard protocol is a novel approach, especially because deep learning (DL) is adopted to improve the outcome. To achieve this, communication behavior signals were simulated according to the communication protocol called MIL-STD-188-141B. Then, the algorithm combing bispectrum estimation of behavior signals with the ameliorated LeNet was adopted. The training time cost of LeNet is low, which is of great practical value. Finally, the experimental results demonstrate that the proposed algorithm is effective for communication behavior recognition purposes.

The main contributions of this work are as follows:

1. A DL method is adopted so that the different communication behaviors of non-collaborative radio stations can be recognized without a standard protocol, which provides a new method for military reconnaissance in the field of electronic countermeasures;
2. In terms of communication behavior signal feature transformation, complex matrices of a signal’s bispectrum estimation are used as the input to the network model;
3. As far as the recognition network model is concerned, LeNet is ameliorated by adjusting the network structure and selecting an appropriate size of convolution kernels. The time cost of training LeNet is discussed because the time cost has to be considered when a recognition network is applied in the battlefield.

The remainder of this paper is organized as follows. Section 2 introduces the generation of communication behavior signals of a short-wave radio station. In Section 3, the proposed algorithm for recognition of non-collaborative radio stations’ communication behaviors is explained in detail. Section 4 presents the experimental results and analysis. Finally, Section 5 concludes the presented work.

## 2. The Communication Behavior Signals of a Short-Wave Radio Station

**Figure 1:**
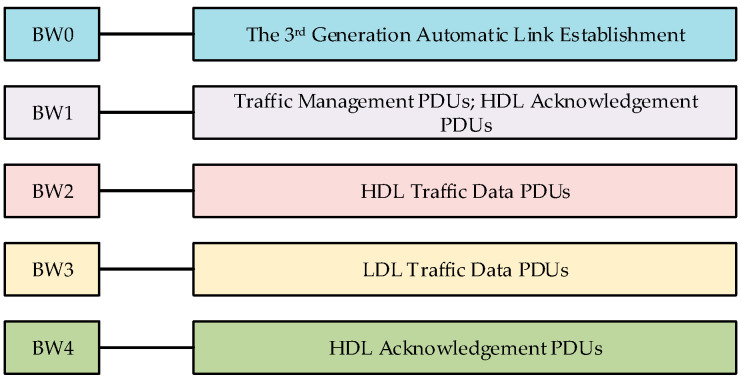
The five kinds of burst waveforms corresponding to different functions in the third-generation short-wave communication protocol. PDU: protocol data unit; HDL: high-rate data link protocol; LDL: low-rate data link.

The third-generation short-wave communication protocol standard (MIL-STD-188-141B) is widely used in the current American military short-wave communication system. The latest short-wave communication systems in Europe and China were also formulated with reference to MIL-STD-188-141B. If a targeted radio station’s communication behavior is recognized from the perspective of the physical layer, it is necessary to analyze the features of signals in the physical layer. MIL-STD-188-141B specifies five kinds of burst waveforms—BW0, BW1, BW2, BW3, and BW4. The five kinds of burst waveforms correspond to different functions in the process of communication, as shown in Figure 1.

In fact, the five functions correspond to five communication behaviors of a radio station, as shown in Figure 1. In this work, the communication behavior recognition of the third-generation short-wave radio station mainly involves recognizing the communication behavior signals corresponding to the five types of burst waveforms identified. The following provides a brief introduction to the simulation, which shows how the burst waveforms (BW0–BW4) are formed. All of the original valid parts of the five types of burst waveforms are represented by randomly generated binary parts.

**Figure 2:**
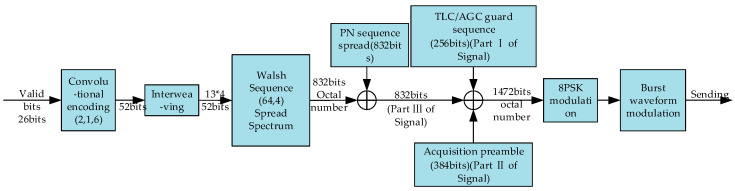
The transmission scheme of BW0.

The data frame of the burst waveform BW0 consists of a transmit-level control (TLC)–automatic gain control (AGC) guard sequence (256 bit phase shift keying (PSK) symbols), acquisition preamble (384 bit PSK symbols), and valid payload (832 bit PSK symbols). The transmission scheme of BW0 is shown in Figure 2.

**Figure 3:**
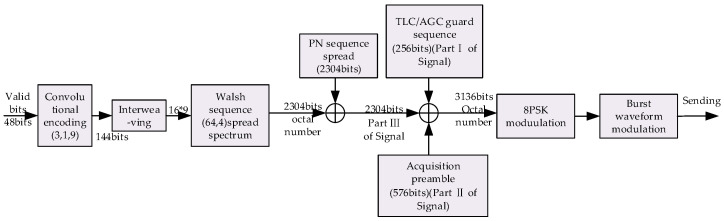
The transmission scheme of burst waveform BW1.

The data frame of the burst waveform BW1 consists of a TLC–AGC guard sequence (256 bit PSK symbols), acquisition preamble (576 bit PSK symbols) and valid payload (2304 bit PSK symbols). The transmission scheme of BW1 is shown in Figure 3.

**Figure 4:**
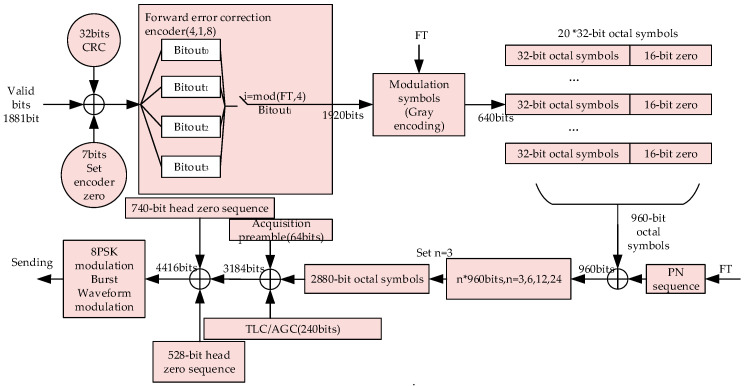
The transmission scheme of burst waveform BW2. FT: forward transmission.

The data frame of the burst waveform BW2 consists of a head zero sequence (704 bit PSK symbols), TLC–AGC guard sequence (240 bit PSK symbols), acquisition preamble (64 bit PSK symbols), valid payload (960 × the number of packet traffics (NumPKTs) bits PSK symbols) tail zero sequence (528 bit PSK symbols), and NumPKTs = 3, 6, 12, or 24. The transmission scheme for BW2 is shown in Figure 4, where FT represents how many forward transmissions have occurred in transmitting the current datagram.

**Figure 5:**
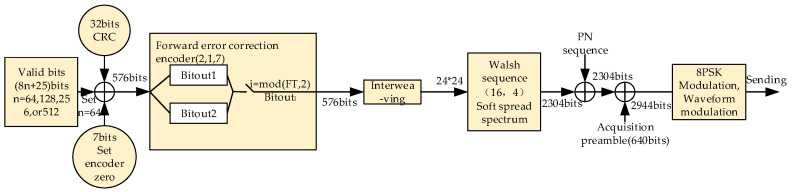
The transmission scheme of burst waveform BW3.

The data frame of burst waveform BW3 consists of an acquisition preamble (640 bit PSK symbols) and valid payload (32 × n + 256 bit PSK symbols), n = 64, 128, 256, or 512. The transmission scheme for BW3 is shown in Figure 5, where FT represents how many forward transmissions have occurred in transmitting the current datagram.

**Figure 6:**
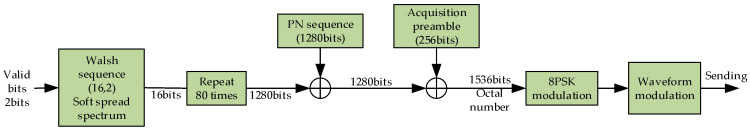
The transmission scheme for burst waveform BW4.

The data frame for burst waveform BW4 consists of an acquisition preamble (256 bit PSK symbols) and valid payload (2 bit PSK symbols). The transmission scheme for BW4 is shown in Figure 6.

**Figure 7:**
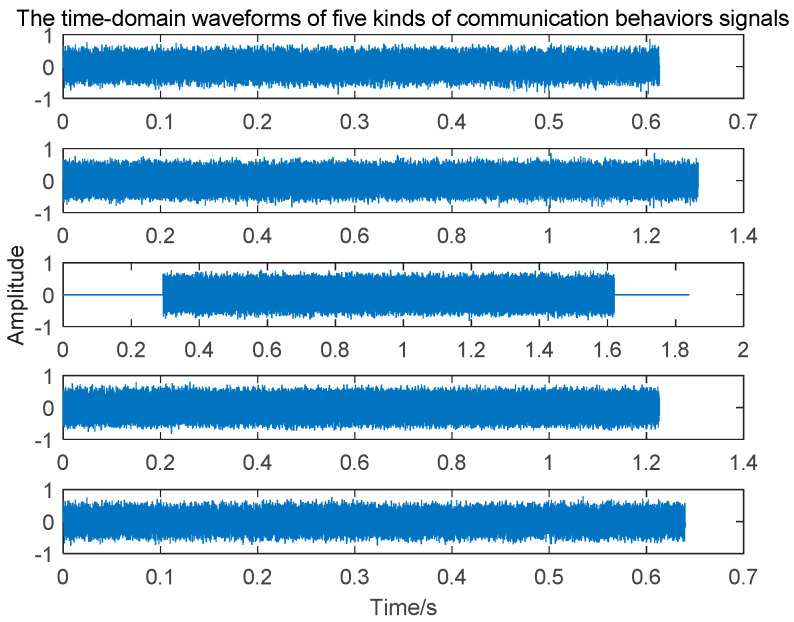
The time domain waveforms of five types of communication behavior signals.

The five burst waveforms were all modulated by 8 PSK. Then, they were up-sampled by interpolation four times and put into an ascending cosine filter. IQ signals were modulated with an 1800 Hz carrier. Finally, the radio signals to be sent were acquired. The parameters of the ascending cosine filter were set as follows: rolling drop coefficient, 0.25; the symbol scope is the length of the sequence, whereby a single symbol was sampled four times. Considering the process of collecting signals in the actual environment, the sampling rate could be set to $2\times (\frac{B}{2}+f_{0})=2\times (\frac{2400}{2}+1800)=6000$ Hz. When actually using sensors to collect signals, we would set the sampling rate to 7500 Hz, avoiding the influence of radio frequency modulation and signal distortion in the process of passing wireless communication channels. Finally, the five types of signals we simulated are shown in Figure 7.

Recognizing different communication behaviors of a short-wave radio station is difficult because there is not much difference in the time domain waveform of each type of communication behavior signal, especially after these communication behaviors signals have passed through the wireless short-wave communication channel. As a non-collaborating party, it is difficult to infer the communication behaviors of a radio station through traditional methods. Therefore, the algorithm described in this work combines the feature transformation of signals and employs deep learning to distinguish the different communication behaviors of a radio station.

## 3. Methods

### 3.1. Bispectrum Analysis of Communication Behavior Signals

This work focuses on the performance of the developed algorithm in terms of recognizing different communication behavior signals in the physical layer. The intercepted signals were simulated by passing the communication behavior signals through the Gaussian white noise channel, as specified in communication protocol MIL-STD-188-141B. Based on these intercepted behavior signals, we conducted an experimental exploration. First, we carried out a signal bispectrum transformation, then we trained the LeNet network, and finally we carried out communication behavior signals recognition. Through the analysis of the simulated physical layer signals, communication behavior recognition of a short-wave radio station was carried out.

The bispectrum function is a two-dimensional Fourier transform of the third-order cumulant. The bispectrum function was used because it is able to transform burst waveforms with different lengths into their features with the same dimensions (Figure 7), allowing behavior signals of different waveforms to be put into the same network model, while other methods cannot obtain features with the same dimensions, for example the Fourier transform, time–frequency transform, and wavelet transform. In addition, the differences in lengths of different waveforms are very small in the real environment, meaning behavior signals cannot be distinguished by the lengths of the waveforms. The bispectrum transform can also retain frequency information and phase information of signals, which means the feature transformation could be effective even if the modulation style of signals is unknown. The bispectrum of the signal, x(t), is defined as:

(1) $$B_{x}(\omega_{1},\omega_{2})=\sum_{\tau_{1}}\sum_{\tau_{2}}C_{3x}(\tau_{1},\tau_{2})e^{-j(\omega_{1}\tau_{1}+\omega_{2}\tau_{2})}$$

where $C_{3x}(\tau_{1},\tau_{2})$ is the third-order correlation function of the signal, defined as:

(2) $$C_{3x}(\tau_{1},\tau_{2})=E\left\{x^{*}(t)x(t+\tau_{1})x(t+\tau_{2})\right\}$$

**Figure 8:**
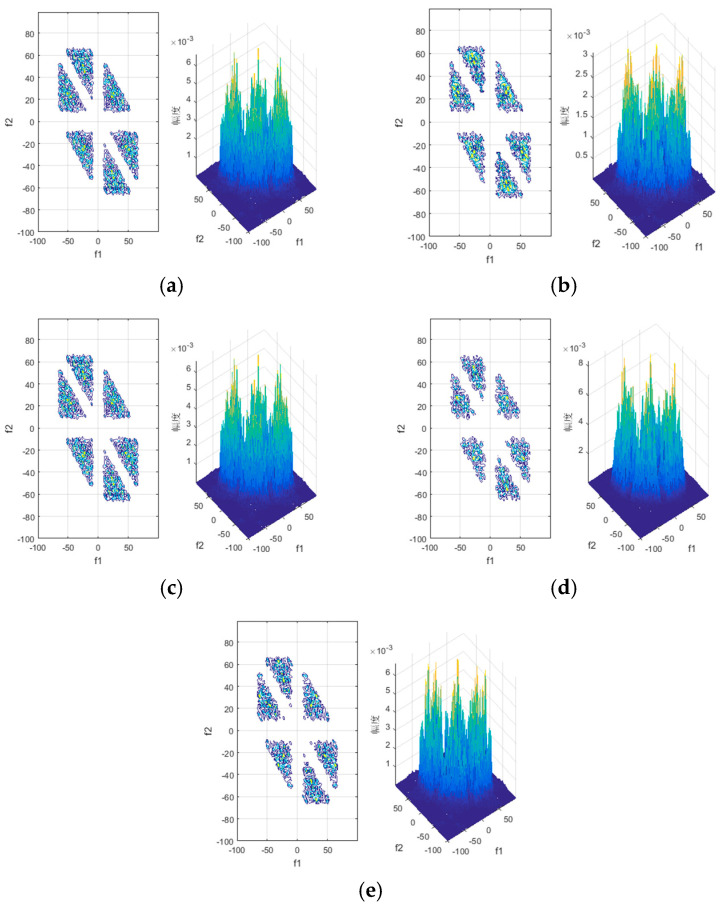
Bispectrum estimations of the five kinds of communication behaviors: (**a**) automatic link establishment behavior; (**b**) traffic management and HDL acknowledgement behavior; (**c**) HDL traffic data behavior; (**d**) LDL traffic data behavior; (**e**) HDL acknowledgement behavior. HDL: high-rate data link protocol; LDL: low-rate data link protocol.

There are parametric and non-parametric methods in the bispectral estimation of a signal. The parameterized method needs to find a model that matches the communication behavior signals acquired by the reconnaissance, which is difficult in a complex electromagnetic environment. Therefore, this work mainly uses the non-parametric method to obtain the bispectrum estimation of the simulated communication behavior signals. According to the non-parametric method of bispectral estimation, when we conduct bispectrum estimation on a one-dimensional signal, we must first divide the signal into *K* segments and then further process each segment. In addition, the parameters in the method are set as follows: the number of sampling points in each segment is set as M=128; the output of the bispectrum estimation is a complex square matrix with a size of 256 × 256. According to the symmetry of the matrix, the complex matrix measuring 128 × 128 at the top right of the square matrix can be used as the deep features of communication behavior signals, and these features are used to train the neural network to recognize communication behaviors. The bispectrum estimations of the communication behaviors are shown in Figure 8.

As can be seen from Figure 8, the bispectrum of the five communication behavior signals are different. According to our analyses of simulated signals, the reason why the differences are small might be that the dimension of the bispectral estimation matrix is too small compared with that of the signals. Hence, the bispectral estimation matrix with dimensions 256 × 256 × 2 cannot retain the full information of the behavior signal’s frequency and phase. Another reason could be that octal-phase modulation was utilized for all communication behavior signals, so the simulated signals only differ in their original binary bits. While this results in small differences among behavior signals, the differences are sufficient to distinguish different communication behavior signals. In addition, to make the differences more obvious, we can expand the dimension of the bispectral estimation matrix, although this could increase the time cost of training the recognition network and reduce the practical value of the proposed algorithm. The slight differences are also the reason why it is difficult to distinguish communication behavior signals of a radio station from the perspective of the physical layer. At present, the frequency information or magnitude information in the bispectral estimation of signals is used separately for recognition using bispectral diagonal slices, a rectangular integral bispectrum, and a selective bispectrum [28,29,30]. In order to retain the subtle features of communication behavior signals and recognize the radio station communication behavior, this work used the improved bispectrum features as the inputs of the recognition network. The magnitude and phase information of the bispectral square matrix were used as the inputs for the recognition network.

To distinguish between such small differences in communication behavior signals, CNN can be used to further extract deep features of signals. Considering the time spent by the real application, the recognition network cannot be too complicated. Thus, the ameliorated LeNet, as a classic CNN, was adopted to recognize the communication behaviors of a short-wave radio station.

### 3.2. LeNet

**Figure 9:**
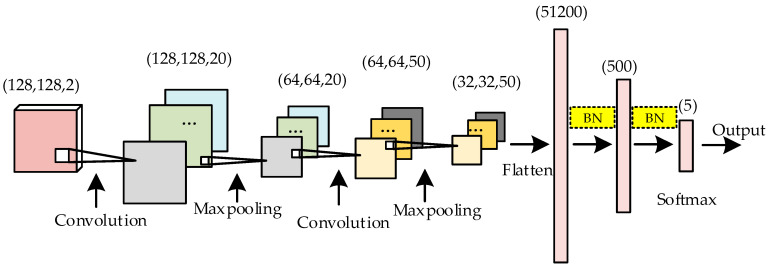
The structure of the ameliorated LeNet. LeNet: a network proposed by LeCun; Maxpooling: max pooling; BN: bath normalization.

LeNet is a classic CNN. Due to its simple architecture and superior performance, LeNet is widely used in image classification, signal recognition, and speech recognition. LeNet includes two modules: a convolutional module and a fully connected module. The structure of the ameliorated LeNet used in this work is shown in Figure 9.

The dimensions of the five types of communication behavior signals of the bispectral estimation matrix measured 256 × 256. According to the symmetry of the bispectral estimation matrix, the upper right side of the matrix was selected and the values of the phase and magnitude corresponding to each element in the matrix were normalized. Finally, a matrix measuring 128 × 128 × 2, which included the frequency information and phase information of the signal, was acquired. The matrix was used as the input of LeNet to train the recognition network. The following improvements were made to LeNet in this work:

(1) We optimized the activation function. The advanced activation function “leaky rectified linear unit (leaky ReLU)” was used instead of the activation function “tanh”, accelerating the gradient descent speed and overcoming the death of neurons;
(2) We optimized the size of the convolution kernel. The size of the convolution kernel was adjusted from (5, 5) to (3, 3) to extract more subtle features;
(3) We used batch normalization (BN). The use of BN in the fully connected layer did not complicate the network but accelerated the training process. BN also reduced the sensitivity of the network model to the learning rate, which had better performance than “dropout”.

### 3.3. Algorithm for Radio Station Communication Behavior Recognition

**Figure 10:**
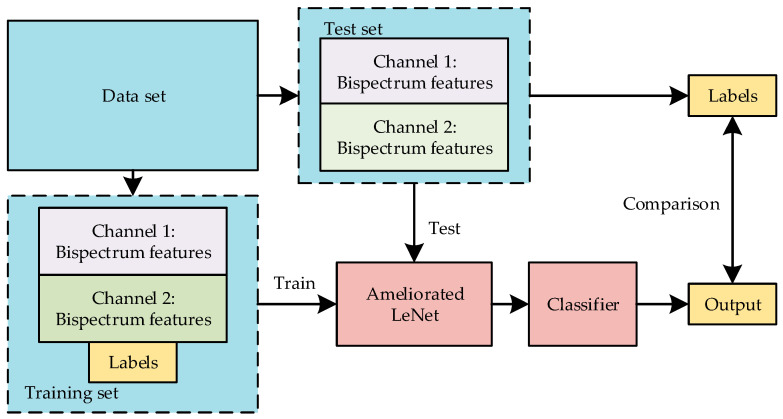
The structure of the proposed algorithm.

The generation of the five kinds of communication behavior signals was in accordance with communication protocol standard MIL-STD-188-141B. To ensure that each burst waveform could fully represent its corresponding communication behavior, the initial valid parts of each burst waveform were randomly generated when a communication behavior signal was simulated. Finally, the communication behaviors signals were generated. There were 1000 samples in each class, totaling 5000 samples of communication behavior signals. All of the communication behavior signals passed through the Gaussian channel before they were used in the recognition algorithm. In this work, the recognition algorithm adopted the basic framework as follows: features transformation, followed by automatic extraction of features, followed by communication behavior recognition. The algorithm model is shown in Figure 10.

The specific steps of the proposed algorithm were as follows:

**Step 1**: Signal features transformation. Calculate the bispectrum of all samples and take the normalized phase and normalized magnitude of each element in the bispectral matrix to form a 2-channel square matrix measuring (128, 128, 2). There were 1000 samples for each label;

**Step 2**: Make data sets. From the 5000 samples generated in step 1, 80% of the samples were randomly selected as the training set and the rest were used as the test set;

**Step 3**: Train the neural network. Firstly, the training set was used to train LeNet and the Adam optimizer was adopted in the training. When the loss function of the network did not change, the training was finished;

**Step 4**: Recognize communication behaviors. The test set was used as the input of the trained LeNet.

## 4. Experimental Results and Analyses

The communication behavior signals used in the experiment were simulated according to MIL-STD-188-141B. Their carrier frequency was 1800 Hz and the sampling rate was 9600 Hz/s. The five types of communication behaviors were automatic link establishment behavior, traffic management and HDL acknowledgement behavior, HDL traffic data behavior, LDL traffic data behavior, and HDL acknowledgement behavior. The bispectral features of the five types of communication behavior signals were different from the conventional picture and text. The feature dimensions were (128, 128, 2); that is, slightly larger than other ordinary pictures, meaning LeNet, which possesses a different internal structure and hyperparameters, needs to be optimized so that it is suitable for the recognition of different communication behaviors. It is also necessary to explore the influence of the Gaussian white noise channel on the recognition performance of the algorithm. In addition, Gaussian noises with different signal-to-noise ratios (SNRs) were added to the five communication behavior signals to imitate the real scene where communication behaviors signals are received by a sensor. The reason why other more advanced classical CNN models were not used here was that their complexity is high and would be unlikely to meet the requirement for rapid reconnaissance. The time required to run various algorithms needs to be explored through experimentation, so that the network that best meets the needs of the application can be chosen. The comparison demonstrates the superiority of the proposed algorithm, which means that the proposed algorithm can better realize the purpose of a short-wave radio station′s communication behavior recognition.

To improve the algorithm in the future, network optimization and algorithm recognition performance experiments were conducted, as well as comparisons with other algorithms.

Experiment environment: Intel(R) i5-4200H CPU; Windows 10; NVIDIA GEFORCE GTX 950M; TensorFlow 1.12.0; Keras 2.2.5.

### 4.1. Network Optimization Experiments

The original LeNet was proposed to solve the problem of simple character recognition. In order to make it more suitable for complex 2-channel bispectral features, the ameliorated network was optimized using the signals dataset with signal-to-noise ratio (SNR) = 10 dB. The optimizations of LeNet mainly refers to two aspects: the location of the batch normalization (BN) layer and the size of the convolution kernel. LeNet benefits from optimization of these aspects because the BN layer can speed up the training of the network, improving the generalization ability of the network and the shuffling of the training samples [31]. A smaller convolution kernel gives more attention to the details of features, which also affects the performance of the network [32]. The global parameters in LeNet were set as follows: batch size = 64; epoch = 5; the number of training samples was 4000; the number of test samples was 1000; the initial learning rate was 0.001.

#### 4.1.1. Experiment on the Location of the BN Layer

The fixed parameter in the experiment was the size of the convolution kernel, set as (5, 5). The variables in the experiments were as follows: The BN layer was added into the fully connected layer, the output layer, and the two convolution layers, expressed as A. The BN layer was added into the two convolution layers, expressed as B. The BN layer was added into the fully connected layer and the output layer, expressed as C. Finally, the BN layer was not added, expressed as D.

**Figure 11:**
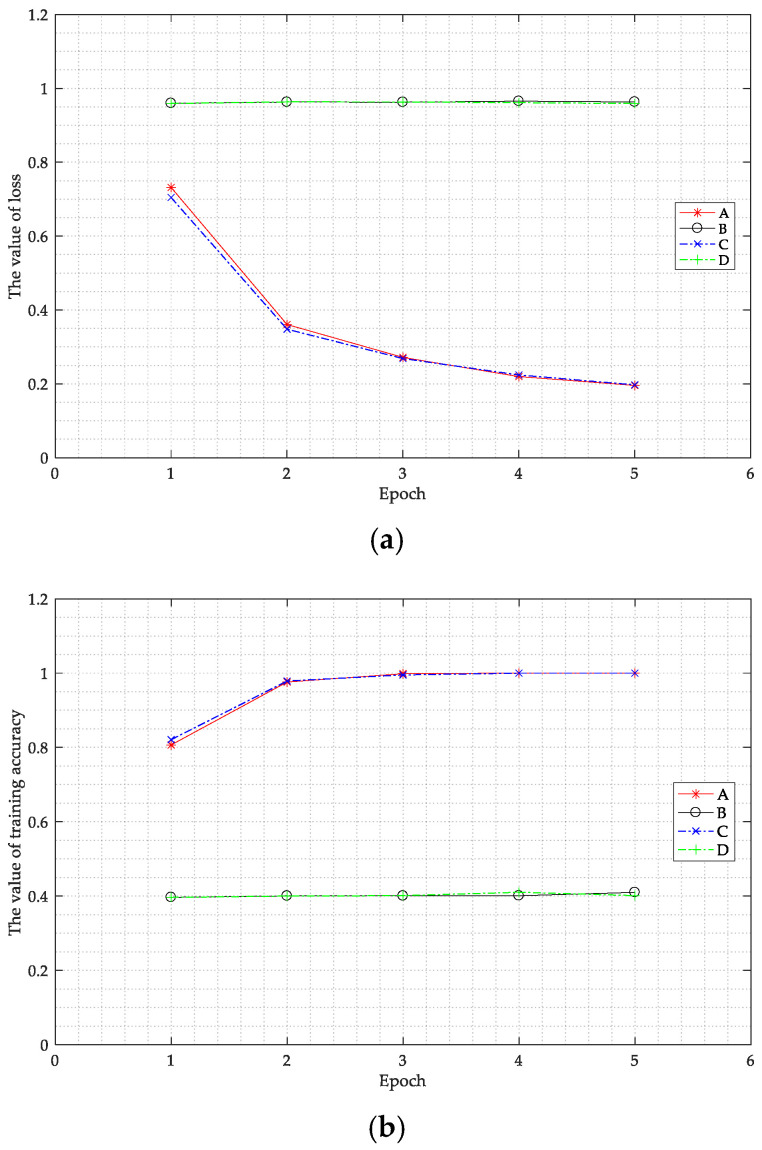
Value of the loss function (**a**) and the value of the training accuracy of the training set (**b**) as the epoch changed when the batch normalization layer was added into different positions. The batch normalization (BN) layer was added into the fully connected layer, the output layer, and the two convolution layers, expressed as A. The BN layer was added into the two convolution layers, expressed as B. The BN layer was added into the fully connected layer and the output layer, expressed as C. Finally, the BN layer was not added, expressed as D.

After training was completed, the training epoch changed. The values of the loss function and training accuracy of the training set are shown in Figure 11.

As shown in Figure 11a, under conditions B and D, the value of the loss function did not change after epoch 2 due to the parameters in the network reaching local optimization. Under conditions A and C, the value of the loss function steadily decreased and the rate of the decrease was basically the same. This shows that the use of the BN layer in the fully connected layer effectively avoids the local optimization of parameters. As shown in Figure 11b, the value of the training accuracy under conditions B and D stabilized at 0.40 after epoch 1. The value of the training accuracy under conditions A and C increased steadily and finally reached about 1.0. The value of loss of C dropped faster than that of A, while the accuracy of C increased faster than that of A. Therefore, the BN layer should be added as C. The experimental results show that when the BN layer is added in the fully connected layer it can accelerate the speed of training the network and improve the performance of the network.

**Figure 12:**
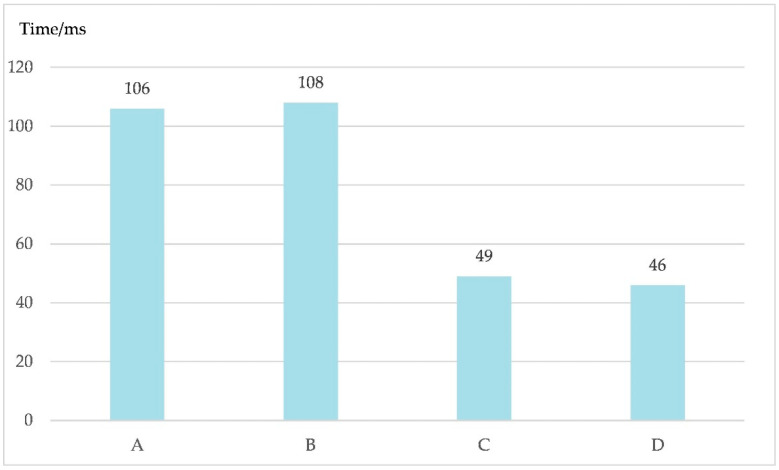
The time spent training each sample when the batch normalization layer was added into different positions. The BN layer was added into the fully connected layer, the output layer, and the two convolution layers, expressed as A. The BN layer was added into the two convolution layers, expressed as B. The BN layer was added into the fully connected layer and the output layer, expressed as C. Finally, the BN layer was not added, expressed as D.

The experiments were conducted under conditions A, B, C, and D, and the corresponding time spent training each sample is shown in Figure 12.

As shown in Figure 12, the corresponding time periods under conditions A and B were 106 ms and 108 ms, respectively. The corresponding time periods under conditions C and D were 49 ms and 46 ms, respectively. The experimental results show that when the BN layer was added into the convolutional layer it greatly increased the time cost of training the network. Meanwhile, adding the BN layer to the fully connected layer did not change the time cost of training the network.

According to Figure 11 and Figure 12, the experimental results also verify that the BN layer can speed up the training of LeNet and avoid local optimization of parameters, as BN has the ability to normalize features and shuffle training samples. Thus, by adding BN layers to the network, the proposed LeNet can learn the deep features of the bispectrum. Considering the time cost of training, adding the BN layer would increase the time cost to a certain extent if added in convolutional layers because BN is not simply a normalization function. The essence of BN is to change the value of variance and the mean, so that the new distribution is closer to the true distribution of data and the non-linear expression ability of the model is also guaranteed.

Overall, the use of the BN layer in the fully connected layer can accelerate the speed of training the network, causing LeNet to avoid falling into local optimization. Moreover, the time cost of training the network barely changes. Therefore, the BN layer was only added into the fully connected layer for subsequent experiments in this work.

#### 4.1.2. Experiment on the Size of the Convolution Kernel

For this experiment, the BN layer was only added to the fully connected layer. The sizes of the convolution kernels were set as (3, 3), (5, 5), (7, 7), and (9, 9), expressed as E, F, G, and H, respectively.

**Figure 13:**
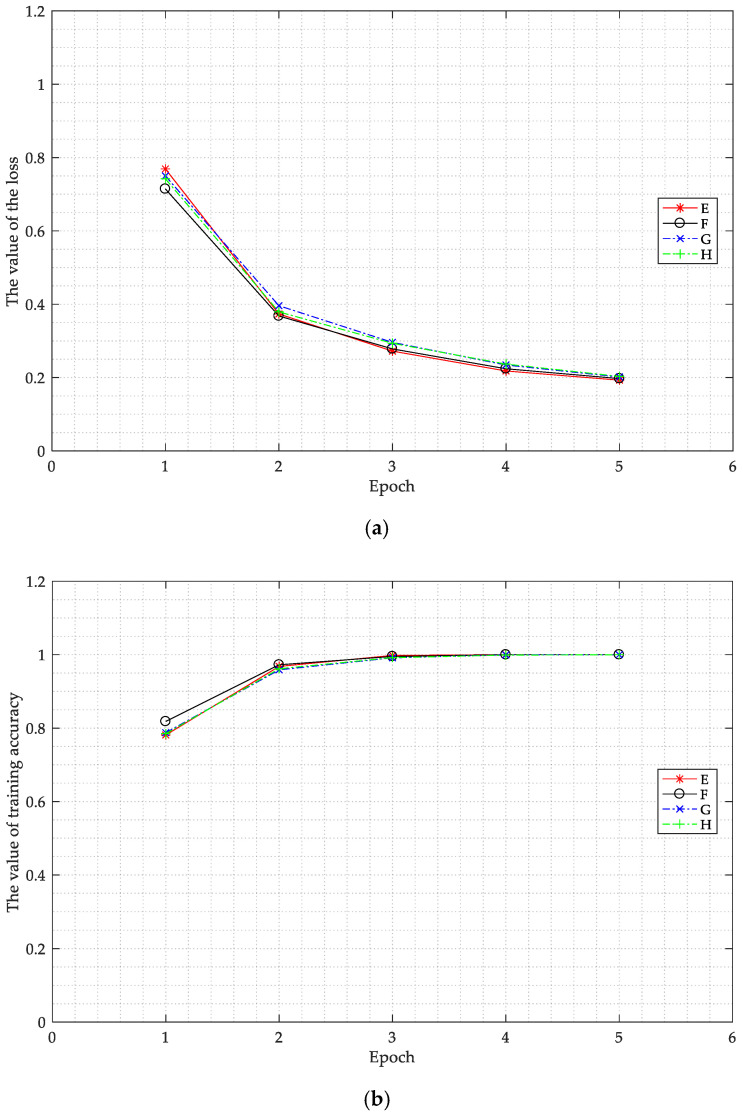
The value of the loss function (**a**) and the value of training accuracy of the training set (**b**) corresponding to the different sizes of convolution kernels as the epoch changed. Kernel sizes (3, 3), (5, 5), (7, 7), and (9, 9) are expressed as E, F, G, and H, respectively.

Figure 13 shows the changes in the values of the loss function and the training accuracy of the training set corresponding to the different sizes of convolution kernels.

As shown in Figure 13a, before epoch 2, in general the smaller the size of the convolution kernel, the slower the value of the loss function decreased. As the epoch increased, the value of the loss function corresponding to smaller sizes of convolution kernels was smaller. When the size of the convolution kernel was larger, the value of the loss function was also larger. As shown in Figure 13b, before epoch 2, the smaller the size of the convolution kernel, the higher the test accuracy of the training set. However, the differences between E, F, G, and H are not clear. If a smaller kernel size is used in the network, the speed of training will be slightly slower, but this will only be a very small difference. More details of the bispectral estimation can be extracted by adopting a smaller kernel size, resulting in improved performance for LeNet. The value of the loss function does not converge as quickly if the size of convolution kernel is smaller. However, as the epoch increases, the features extracted by a smaller kernel can better reflect the essence of the samples belonging to one class.

**Figure 14:**
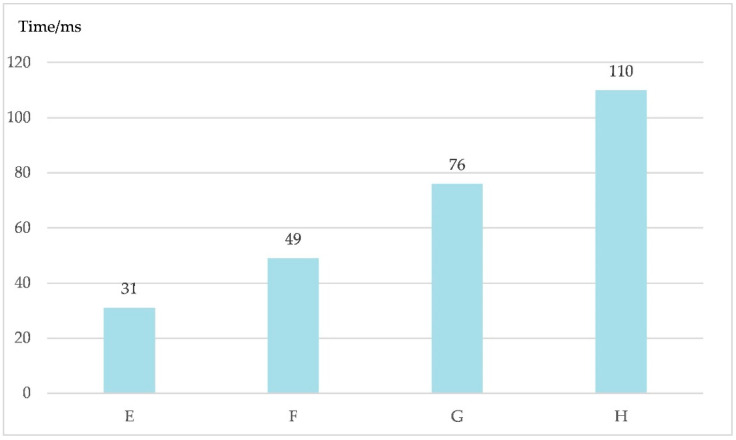
Time periods spent training each sample with different convolution kernel sizes of (3, 3), (5, 5), (7, 7), and (9, 9), expressed as E, F, G, and H, respectively.

The time periods spent training each sample under conditions E, F, G, and H are shown in Figure 14.

As shown in Figure 14, as the size of the convolution kernel increases, the time spent training each sample gradually increases from 31 ms to 110 ms. Combined with Figure 13, it is obvious that the smaller the size of the convolution kernel, the less time is spent training the network. Moreover, a smaller convolution kernel can better reflect the essential differences between communication behavior signals. Therefore, the size of the convolution kernel adopted in LeNet was set as (3, 3) for subsequent experiments.

According to all of the experiments in Section 4.1, the different internal structures and the kernel size in LeNet have important impacts on the performance of the algorithm. We gradually optimized the network by fixing the size of the convolution kernel and selecting the appropriate structure through experiments, and then with the appropriate structure we chose the optimal size of the convolution kernel. Considering the rapid response and performance of LeNet in practical applications, the original LeNet was improved here by adding the BN layer into the fully connected layer and setting the size of the convolution kernel to (3, 3).

### 4.2. Experiments on the Recognition Performance of the Algorithm

He standard protocol MIL-STD-188-141B is used widely in short-wave communication systems. The protocol standard stipulates that the Gaussian noise channel can be used as a wireless communication channel to verify the performance of the communication system. Therefore, the influence of Gaussian noise with different SNRs on the performance of LeNet should be explored. First, 5000 simulated signals belonging to five signal types of communication behavior passed through different Gaussian noise channels with SNR = 0 dB, 5 dB, 8 dB, 10 dB, and 15 dB. Then, the magnitude square matrix and the phase square matrix of each signal’s bispectral estimation were labeled by the corresponding category of the signal. In total, 1000 samples belonging to each category were simulated. The magnitude square matrix and the phase square matrix of each signal were treated together as one sample so that 5000 samples were generated. Finally, 4000 samples were randomly selected to train the ameliorated LeNet and the remaining 1000 samples were used as the test set.

The fixed parameters were as follows: the batch size was fixed at 64; there were 10 epochs; the initial learning rate was 0.001; and the size of the convolution kernel was (3, 3).

**Figure 15:**
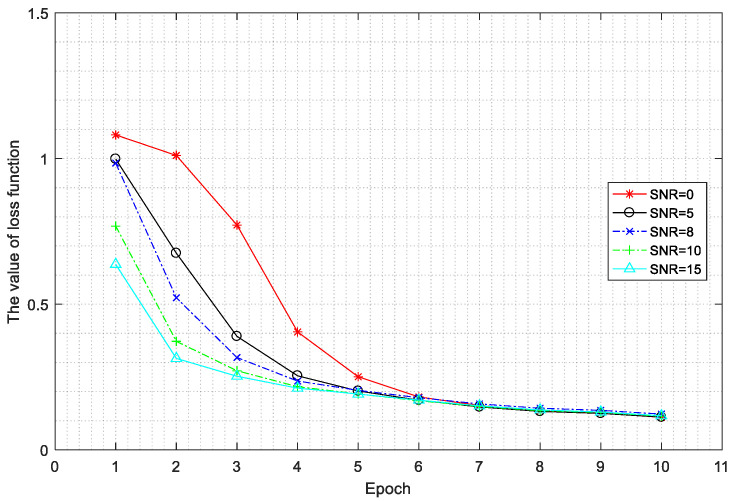
The value of the loss for different signal-to-noise ratios (SNRs) over training epochs.

The change in value of the loss function for the different SNRs over the training epochs is shown in Figure 15.

**Figure 16:**
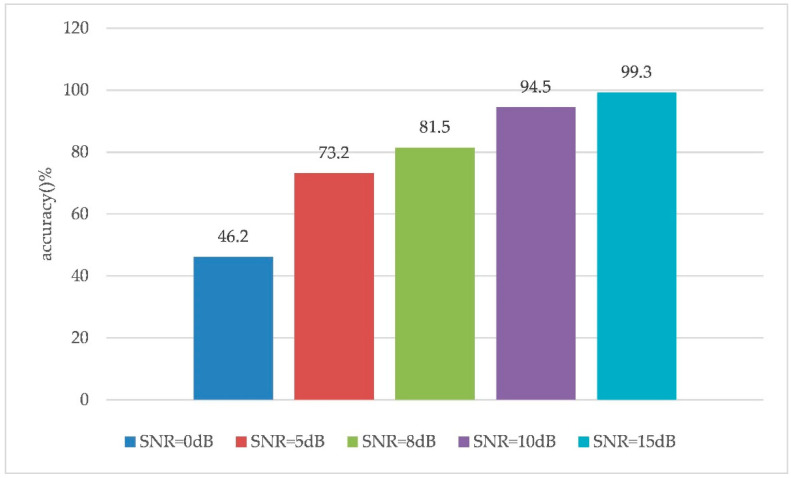
The test accuracy of the test set under different signal-to-noise ratio (SNR) conditions.

Figure 15 shows that the higher the SNR, the faster the value of the loss function decreases, which means the faster the algorithm converges. The value of the loss function gradually stabilizes at epoch 7. The test accuracy of the trained network on the test set is shown in Figure 16.

As shown in Figure 16, with the improvement of the quality of the wireless short-wave communication channel, the recognition accuracy of the proposed algorithm in this work gradually improves. When the SNR values were 0 dB and 5 dB, the test accuracy values of the test set reached 46.2% and 73.2%, respectively. At an SNR was greater than 8 dB, the algorithm had good recognition performance. The test accuracy values were 81.5%, 94.5%, and 99.3% when the SNR was 8 dB, 10 dB, and 15 dB, respectively. At low SNRs, the recognition performance of the algorithm still needs to be improved. Nonetheless, we achieved the recognition of different communication behaviors without a communication protocol standard, which is significant. In real applications of the algorithm, the de-noising technology can be used to process the intercepted communication behavior signals, after which the proposed algorithm can be adopted to recognize a short-wave radio station’s behaviors by utilizing the processed signals.

In order to explore the influence of the number of samples used to train network on the classification accuracy, the signal data set with SNR being 10 dB was utilized to conduct following experiments. Finally, the recognition accuracy of the proposed algorithm is shown in Table 1.

Table 1 shows that the more samples that were used to train network, the higher the recognition accuracy of the proposed algorithm. When the numbers of training samples were 500, 1000, 2000, 3000, and 4000, the recognition accuracy values were 36%, 45.5%, 61.7%, 80.1%, and 94.5%, respectively. Moreover, when the number of training samples was greater than 3000, the recognition accuracy was greater than 80%, indicating that 3000 samples were needed for the bispectrum to reveal the signal features.

### 4.3. Comparison Experiments

With regards to neural network selection for the recognition of a radio station’s communication behaviors, here simple LeNet was used to extract the deep features of the samples and then the Softmax classifier was utilized to complete the recognition of different communication behaviors. At present, there are other more advanced CNN models, such as AlexNet, GoogleNet, and ResNet. In the field of computer vision, the recognition performance of these networks is generally higher than LeNet. However, in the field of radio reconnaissance, the complexity of these networks incurs a high time cost to train the network, which is a significant drawback. Thus, we carried out some experiments to explore the performance and time costs for the adoption of different CNN models to recognize communication behavior signals. In addition, these experiments can explain the reason why the ameliorated LeNet was adopted for this work. Finally, the performance of the proposed algorithm was compared with some traditional radio signal recognition algorithms.

The time cost of training the ameliorated LeNet was compared with the classic LeNet, classic AlexNet, classic GoogleNet, and classic ResNet. The signals with SNR = 10dB were used in the experiment and the data set was generated by the magnitude square matrix and phase square matrix of these signals’ bispectral estimations.

The fixed parameters in experiments were as follows: the batch size was 64; there were five epochs; the initial learning rate was 0.0001; the training and test sets consisted of 4000 and 1000 samples, respectively.

**Figure 17:**
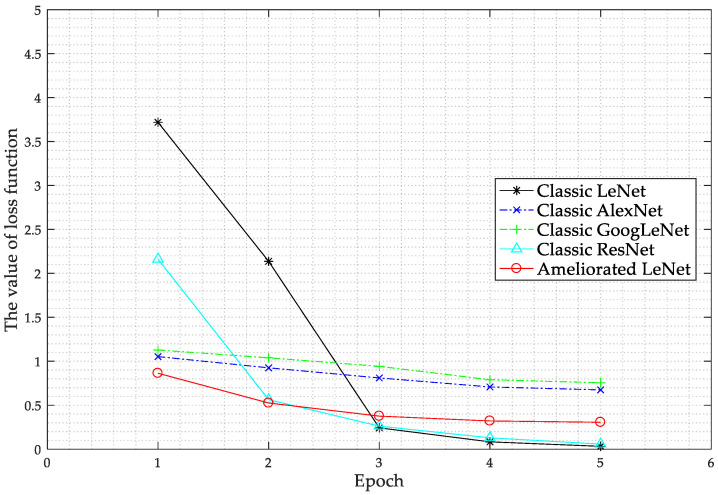
The value of loss function changing over epochs for the different network models compared.

The values of the loss function changes during the training of each network are shown in Figure 17.

**Figure 18:**
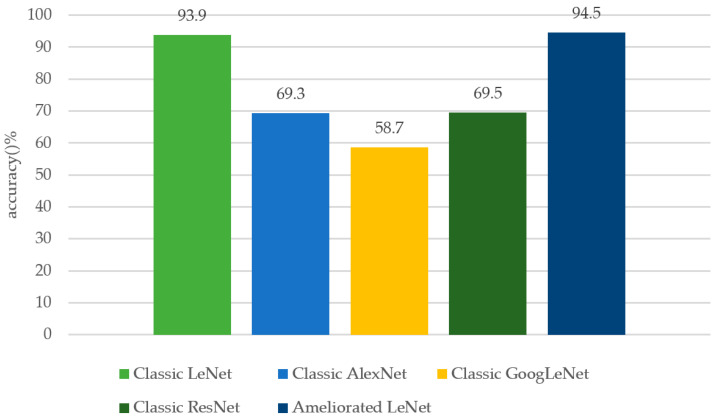
The test accuracy of every network on the test set at epoch five.

As shown in Figure 17, after the second epoch, the loss of the ameliorated LeNet, classic AlexNet, and classic GoogLeNet is very small, and then the loss declines more slowly. Before the third epoch, the loss of the classic LeNet and classic ReNet declines rapidly and then the loss function declines slowly. The loss of every network model tends to be stable by epoch five, although local optimization may occur due to the different internal structures of each network. The test accuracy of every network on the test set at epoch five is shown in Figure 18.

As shown in Figure 18, the classic LeNet and ameliorated LeNet, which have simpler structures, have the best performance. As the complexity of the networks increases, the other networks more easily fall into the trap of local optimization; thus, the recognition performance of these networks may deteriorate. Combining Figure 17 and Figure 18, we know that every network becomes stable after epoch five because the loss function changes very little. Thus, the test accuracy of the networks at epoch five represents the final performance. In addition, the complex matrix of the bispectral estimation was used to train the network, but the differences among communication behavior signals were not very obvious, as Figure 8 shows, so the features to be extracted by networks might not differ greatly. Hence, the simple LeNet had better performance than other networks with more complicated structures. Of course, there is more work needed on this topic, as the research on this subject has just begun.

**Figure 19:**
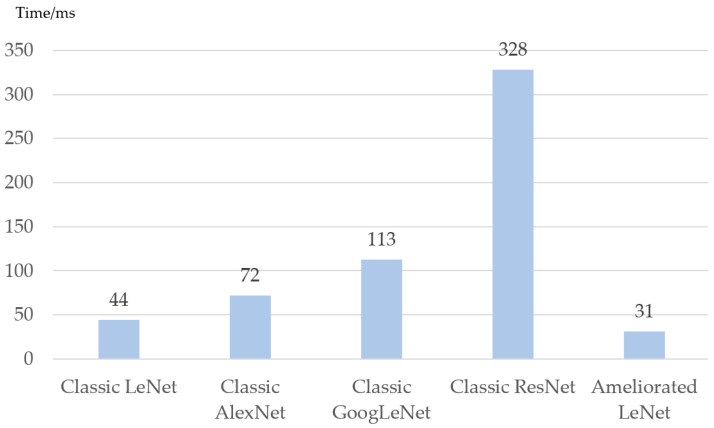
The time spent training each sample for the different networks studied.

In practical applications, the time cost of training the network will be an important issue, which was also the original motivation for choosing LeNet rather than other networks. The time spent training each sample corresponding to every network in Figure 17 and Figure 18 is shown in Figure 19.

Figure 19 shows that the time cost of the ameliorated LeNet is lower than other networks. It takes about 31 ms to train each sample for the ameliorated LeNet. The time to train ResNet is ten times higher, which means the ReNet is not suitable for practical applications.

**Figure 20:**
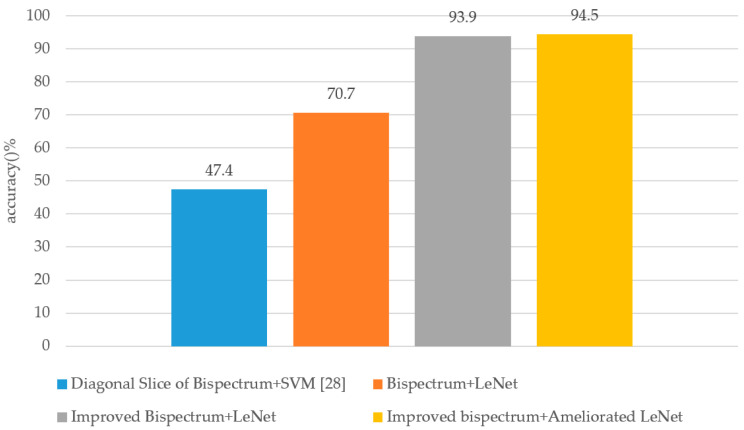
The recognition accuracy of different algorithms. Bispectrum: the magnitude information of bispectral estimation, which is used widely in traditional signal recognition. Improved bispectrum: the magnitude information and the phase information of the bispectral estimation utilized together. LeNet: the original LeNet.

Finally, some traditional algorithms were adopted to recognize short-wave radio station communication behavior signals. Yuan et al. [29] and He et al. [30] adopted respectively rectangular integral bispectrum and selected bispectra only using the magnitude matrix of the bispectral estimation, so their methods are treated as a traditional method called the “magnitude matrix of the bispectrum”. Another traditional method used in the experiment was the diagonal slice of the bispectrum [28]. The performance of the different algorithms is shown in Figure 20.

Figure 20 shows that the proposed algorithm had better performance than other traditional algorithms. The accuracy of the proposed algorithm reached up to 94.5%, which was 47.1%, 23.8%, and 0.6% higher than that of the diagonal slice of bispectrum+support vector machine (SVM), bispectrum+LeNet, and improved bispectrum+LeNet, respectively. The recognition accuracy of the improved bispectrum+LeNet was 93.9%, while that of bispectrum+LeNet was 70.7%, which means the proposed complex matrix of the bispectrum estimation can retain more features of communication behavior signals. Moreover, the recognition accuracy of the proposed algorithm was 0.6% higher than that of the improved bispectrum+LeNet, which shows that our work to ameliorate original LeNet was successful.

According to the experiments in Section 4.3, the time cost of the proposed algorithm was lower and the recognition accuracy of the proposed algorithm was higher than other algorithms. The proposed algorithm meets the needs of practical applications.

## 5. Conclusions

An algorithm based on bispectral features and ameliorated LeNet was proposed in this study of short-wave radio station communication behavior recognition. Compared with traditional methods, the proposed algorithm does not require the communication protocol standard of non-collaborative organizations. For this study, communication behavior signals were simulated according to communication protocol MIL-STD-188-141B. In real environments, we can only obtain behavior signals collected by sensors, so we added Gaussian noise to simulated signals. Thus, the communication behavior signals passing through wireless communication channels of different qualities were acquired. In terms of the preprocessing of signals, the bispectral features can preserve the information of a signal’s frequency and phase and can transform the five types of burst waveforms of different lengths into a square matrix with the same dimensions, which makes it easier to input behavior signals into the network model. In terms of the recognition network, CNN has a strong capability for learning deep features, so an ameliorated LeNet was adopted here. The structure of LeNet was optimized by a series of experiments, which made LeNet more suitable for communication behavior signal recognition. The performance of the proposed algorithm was superior to the algorithm based on the bispectral diagonal slice and the algorithm based on more complex CNN models. The high recognition accuracy and low time cost of the proposed algorithm showed that it is of high practical value in the field of electronic reconnaissance. We can use sensors to capture signals from non-cooperative organizations and then analyze the communication intent represented by the signals.

In the future, the proposed algorithm can be improved, for example through more efficient extraction of features and by optimizing the selection of the neural network. In fact, we did not thoroughly explore the impacts of each network’s structure and hyperparameters on recognition performance. Moreover, the communication behavior signal should be collected by sensors in the battlefield and then used to verify the effectiveness of the proposed algorithm. This work provides new ways to analyze a non-collaborative radio station’s topological structure and tactical status, even without a standard protocol.

## Figures and Tables

**Table 1:** The recognition accuracy corresponding to different numbers of training samples (SNR = 10 dB).

The number of samples	500	1000	2000	3000	4000
Recognition accuracy (%)	36.0	45.5	61.7	80.1	94.5
